# Increased transcription in hydroxyurea-treated root meristem cells of *Vicia faba*

**DOI:** 10.1007/s00709-012-0402-x

**Published:** 2012-04-15

**Authors:** Konrad Krajewski, Justyna Teresa Polit, Janusz Maszewski

**Affiliations:** Department of Cytophysiology, Institute of Physiology, Cytology and Cytogenetics, University of Lodz, ul. Pomorska 141/143, 90-236 Łódź, Poland

**Keywords:** 5-Ethynyl uridine, Transcription, Acetylation, RNA polymerase, RPB1, *Vicia faba*

## Abstract

Hydroxyurea (HU), an inhibitor of ribonucleotide reductase, prevents cells from progressing through S phase by depletion of deoxyribonucleoside triphosphates. Concurrently, disruption of DNA replication leads to double-strand DNA breaks. In root meristems of *Vicia faba*, HU triggers cell cycle arrest (preferentially in G1/S phase) and changes an overall metabolism by global activation of transcription both in the nucleoplasmic and nucleolar regions. High level of transcription is accompanied by an increase in the content of RNA polymerase II large subunit (POLR2A). Changes in transcription activation and POLR2A content correlate with posttranslational modifications of histones that play a role in opening up chromatin for transcription. Increase in the level of H4 Lys5 acetylation indicates that global activation of transcription following HU treatment depends on histone modifications.

## Introduction

Cell cycle transitions throughout interphase and mitosis are regulated by sophisticated metabolic pathways comprising diverse proteins. To ensure DNA integrity and correct functioning of important cellular processes, such as replication or mitotic division, cells take advantage of their evolutionary developed mechanisms called cell cycle checkpoints. Genotoxic stress caused by a variety of potential stressors (e.g., ultraviolet light, ionizing radiation, chemicals that affect DNA integrity) combined with disruption of checkpoint control functions may have a destructive impact on cellular functioning (Shackelford et. al. 1999; Bartek and Lukas 2001; Rybaczek and Kowalewicz-Kulbat 2011). Adequate response to DNA damage is possible owing to the presence of specific sensor kinases that constitute upstream factors to their effectors. Multidimensional character of cell cycle checkpoints allows not only to block phase-to-phase transitions but also, if necessary, to activate diverse genes and to trigger DNA repair processes (Jackson 2002; Yang et al. 2004). Replication fork stalling or DNA lesions are detected by two sensor kinases (PIKK family members), ATM (ataxia telangiectasia-mutated) and ATR (ATM and Rad 3-related) and their downstream factors—Chk1 and Chk2 kinases. Consequently, the activity of the latter proteins drives inhibitory phosphorylation of Cdc25 phosphatases, which become unable to activate cyclin and Cdk complexes, ultimately resulting in cell cycle arrest (Abraham 2001; Ricaud et al. 2007; McNeely et al. 2010). In turn, repair factors involved in homologous recombination or non-homologous end-joining are recruited to DNA lesions, owing to gamma phosphorylation of H2AX histones by sensor kinases (Rogakou et al. 1998; Hanakahi et al. 2000; Bassing et al. 2002). Molecular components of cell cycle checkpoints are well known, yet mutual relationships between these factors and other cellular proteins are still not clear.

Hydroxyurea (HU) is a well-known inhibitor of ribonucleotide reductase (RNR) which, by transformation of ribonucleotides to deoxyribonucleoside triphosphates, plays a fundamental role in establishing balanced quantities of precursors required for DNA synthesis and DNA repair systems (Roa et al. 2009; Koç et al. 2004; Alvino et al. 2007). Inhibition of RNR activity prevents cells from progressing through S phase by enhanced production of replication intermediates, including long ssDNA regions and inappropriate processing of the reversed forks, which leads to dsDNA breaks (DSB; Sogo et al. 2002). Moreover, HU triggers changes in an overall cellular metabolism resulting in an enhanced expression of diverse genes, e.g., γ-globin (Watanapokasin et al. 2006), adult β-globin in HeLA cells (Zhang et al. 2001), or *SMN2* (survival motor neuron) in lymphoblastoid cell lines (Grzeschik et al. 2005). There are only few reports considering the effect of HU on transcription activation, and the molecular bases of this phenomenon are poorly understood. The results presented in this work indicate that in root meristems of *Vicia faba*, HU not only leads to cell cycle arrest but also triggers changes in cellular metabolic state manifested by global transcription activation correlated with an increased content of RNA polymerase II and histone H4 Lys5 acetylation (H4K5Ac).

## Material and methods

### Material

Seeds of *V. faba* subsp. *minor* var. *Nadwiślański* were sown on wet filter paper in Petri dishes and germinated for 3 days at room temperature in darkness. For experiments, seedlings with roots ranging from 1.5 to 2.0 cm in length were selected and incubated in water or 2.5 mM HU for 24 h.

### Chemical agents

Hydroxyurea, pararosaniline, ethylenediaminetetra acetic acid, HEPES, phenylsulfonyl fluoride, dithiothreitol, Protease Inhibitor Cocktail (P-9599), Coomassie blue, DABCO (1,4-diazabicyclo [2.2.2] octane), and 4′,6-diamidino-2-phenylindole (DAPI) were supplied by Sigma, Triton X-100 and pectinase from *Aspergillus niger* by Fluka, cellulose Onozuka R-10 from *Trichoderma viride by* SERVA, pectolyase Y-23 by ICN, and acetic acid by Chempur. Click-iT® RNA Alexa Fluor® 488 Imaging Kit for visualization of RNA transcripts, NuPAGE® Novex® 4–12 % Bis-Tris gel, NuPAGE® Novex® 3–8 % Tris-Acetate gel, polyvinylidene fluoride membrane (0.2-μm pore size), and Chromogenic Western Blot Immunodetection Kit were supplied by Invitrogen. P-PER Plant Protein Extraction Kit was obtained from Pierce (Rochford, USA). Other chemicals were obtained from POCH S.A.

### Feulgen staining and cytophotometry

Apical fragments of roots (1.5 cm long) were fixed in Carnoy’s mixture (ethanol/glacial acetic acid; 3:1, *v*/*v*) for 1 h. Following fixation, roots were rinsed three times in 96 % ethanol, rehydrated (70–30 % ethanol, distilled water), hydrolyzed in 4 M HCl (1.5 h), and stained with Schiff’s reagent (pararosaniline). After 1-h staining, roots were rinsed in SO_2_–water (three times) and then in distilled water. Root tips (1.5 mm long) were cut off and squashed in a drop of 45 % acetic acid onto slides using the dry ice method. After removing cover slips, slides were plunged into 70 % ethanol, air dried, and mounted in Canada balsam. Nuclear DNA content was evaluated by means of microdensitometry using a Jenamed 2 microscope (Carl Zeiss, Jena, Germany) with the computer-aided Cytophotometer v1.2 (Forel, Lodz, Poland). Feulgen-stained cell nuclei were measured at 565 nm.

### Chemical labeling and detection of RNA transcripts

Click-iT® RNA Alexa Fluor® 488 Imaging Kit was used for visualization of RNA transcripts (Jao and Salic 2008). Root fragments (1.5 cm) were cut off and transferred to 2 mM water solution of 5-ethynyluridine (5-EU; control) or to the mixture of EU and HU, in dark. After 1.5-h labeling, 1.5-mm apical root parts were fixed in phosphate-buffered saline (PBS)-buffered 4 % paraformaldehyde (4°C; pH 7.4) for 45 min. For maceration, meristems were rinsed twice in PBS and transferred for 45 min to the citrate-buffered mixture (pH 5.0; 40°C) containing 2.5 % pectinase from *A. niger*, 2.5 % cellulose Onozuka R-10 from *T. viride*, and 2.5 % pectolyase Y-23. Next, the root tips were rinsed twice in cold PBS, squashed onto microscope slides (Polysine™, Menzel-Gläser) in a drop of distilled water, and placed on dry ice. After 10 min, cover slips were removed, and slides were washed with PBS, distilled water, and air dried. Then, the macerated cells were permeabilized with 0.5 % Triton X-100 for 15 min. Sites of EU incorporation were detected using Click-iT® reaction cocktail consisting of components prepared according to the vendor’s manual. Incubation was performed at room temperature for 60 min. After that time, slides were washed in Click-iT® reaction rinse buffer and PBS. Cell nuclei were stained with DAPI (15 μM) for 15 min and then washed in PBS. Specimens mounted in PBS/glycerol mixture (9:1) containing 2.5 % DABCO were photographed under the Eclipse E600W microscope. For Alexa Fluor® 488 DM 505 filter (excitation wavelength, 465–495 nm) and for DAPI DM 400 filter (excitation wavelength, 340–380 nm) were used.

### Immunodetection of POLR2A and histone H4 Lys5 acetylation

Roots were fixed in PBS-buffered 4 % paraformaldehyde (4°C; pH 7.4) for 45 min (maceration performed as for detection of RNA transcripts) or for 20 min (isolation of cell nuclei). For isolation of nuclei, fixed meristems were rinsed twice in PBS and squashed between two slides in a drop of PBS. Fractions of isolated nuclei were harvested on microscope slides and air dried.

Immunodetection of acetylated Lys5 was performed using macerated root tips, while large subunit of RNA polymerase II (POLR2A) was visualized in fractions of isolated cell nuclei. The specimens were preincubated in the blocking buffer [5 % bovine serum albumin (BSA), 0,3 % Triton X-100, PBS] and then incubated in primary anti-acetyl-histone H4 (Lys5) antibodies (1:400, Cell Signaling) dissolved in antibody dilution buffer (1 % BSA, 0,3 % Triton X-100, PBS), or in anti-C-terminal POLR2A antibodies (1:75, Sigma) dissolved in the antibody dilution buffer (1 % BSA, PBS), in both cases overnight at 4°C. After that, slides were washed in PBS and then incubated with secondary FITC-conjugated anti-rabbit IgG (whole molecule; 1:350, Sigma) dissolved in the antibody dilution buffer (1 % BSA, 0.3 % Triton X-100, PBS), at room temperature for 90 min. Nuclear DNA was stained with DAPI (15 μM) for 15 min and then washed in PBS. The specimens were mounted and observed as described previously.

### Western blotting

P-PER Plant Protein Extraction Kit supplemented with Protease Inhibitor Cocktail was used for total protein extraction. In turn, the method presented by Busk and Pages (1997) was applied to obtain nuclear proteins lysates. Whole-cell protein extracts was fractionated on NuPAGE® Novex® 4–12 % Bis-Tris gel and nuclear protein lysates on NuPAGE® Novex® 3–8 % Tris-Acetate gel and then both blotted onto polyvinylidene fluoride membrane (0.2-μm pore size). POLR2A was detected with the rabbit polyclonal anti-C-terminal domain antibodies diluted to 1:750 and goat anti-rabbit IgG antibody conjugated with alkaline phosphatase, using the Chromogenic Western Blot Immunodetection Kit.

### Fluorescence intensity measurements and statistical analysis

Quantitative measurements of fluorescence were based on microscopic photographs, converted to grayscale. Computer-aided Cytophotometer v1.2 (Forel, Lodz, Poland) was used for microdensitometry analysis of fluorescence intensity. Cells were assigned to successive stage of interphase on a basis of fluorescence intensity obtained from DAPI-stained nuclei. Median values of fluorescence intensity following EU incorporation and H4 Lys5 immunodetection were estimated basing on 150 to 250 individual measurements of nuclei for each phase of the cell cycle, both in the control and HU-treated root meristems. In turn, median intensities of POLR2A immunofluorescence of nuclear regions were evaluated following 40–60 individual measurements per experimental series.

Statistical analysis was performed using Statistica 9.1 PL software. Differences between groups were assessed by Kruskal–Wallis test. A *p* value smaller than 0.05 was considered as statistically significant.

## Results and discussion

### Hydroxyurea triggers the G1/S phase cell cycle arrest in root meristems of *V. faba*

**Fig. 1:**
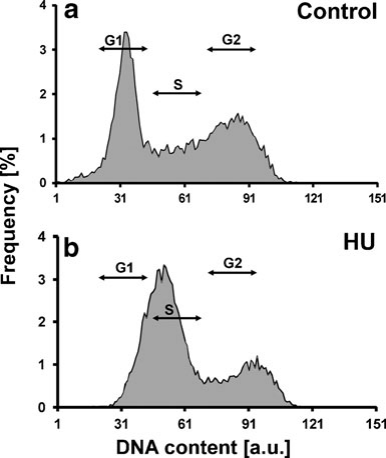
Frequency distribution (percentage) of nuclear DNA contents in the control (**a**) and in HU-treated cells (**b**); nuclear DNA Feulgen staining; *a.u.* arbitrary units

Cytophotometric studies showed that in root meristems of *V. faba* treated with 2.5 mM HU for 24 h, cells accumulated preferentially in G1- and S phase (Fig. 1a, b). Similar results were obtained by Dolezel et al. (1999) following 18-h incubation in 2.5 mM HU. These data seem to be also consistent with those indicating G1/S phase arrest following HU treatment in animals (Borel et al. 2002; Lentini et al. 2006; Kaida et al. 2011) and in some plants, such as *Arabidopsis thaliana* (Culligan et al. 2004) or *Datura inoxia* (Conia et al. 1990). However, Rybaczek et al. (2008) revealed G2 arrest in root meristems of *V. faba* treated for 24 h with 2.5 mM HU. It should be taken into account that lots of factors may have impact on induction of phase-specific cell cycle arrest, especially when one considers that HU may not completely block replication and the cell cycle can still move forward. Occurrence of micronuclei indicated that some cells still continued cell cycle progression and preserved the ability to enter aberrant mitotic division in spite of blocked or slowed down DNA replication (data not shown). Moreover, cells of *Allium cepa* blocked by an intra-S checkpoint, activated in response to HU, were able to complete both their DNA synthesis and post-replication repair (Pelayo et al. 2003).

### Hydroxyurea brings about changes in the dynamics of transcription and RNA polymerase II content

**Fig. 2:**
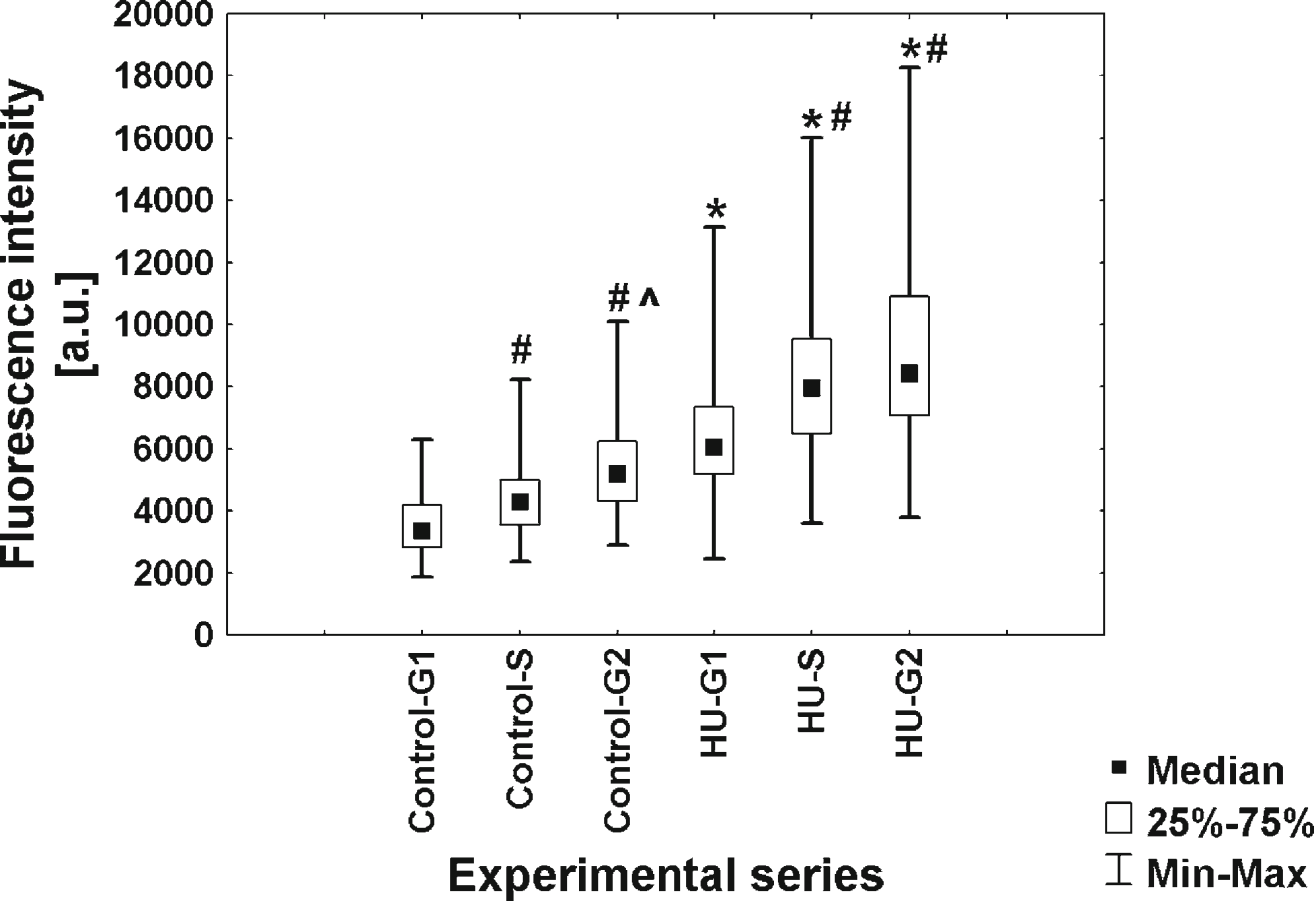
Median fluorescence intensity (*a.u.* arbitrary units) in the nucleoplasmic region evaluated following 5-EU incorporation into root tip cells from seedlings incubated in H_2_O and HU; successive phases of the cell cycle in the control plants denoted as *Control-G1*, *Control-S*, and *Control-G2* while in seedlings incubated in 2.5 mM HU (24 h) denoted as *HU-G1*, *HU-S*, and *HU-G2*. Statistical significance (Kruskal–Wallis test): *p < 0.001 control-G1/HU-G1, control-S/HU-S, control-G2/HU-G2; ^#^*p* < 0.001 control-G1/control-S, control-G1/control-G2, HU-G1/HU-S, HU-G1/HU-G2; ^^^*p* < 0.001 control-S/control-G2

To evaluate the intensity of transcription in root meristem cells of *V. faba*, a new technique based on 5-ethynyluridine (EU) incorporation was applied. This method allows to detect newly synthesized RNA molecules by cycloaddition reaction, without applying immunocytochemical procedures. Quantitative results are shown as median fluorescence intensity. Although fluorescence measurements do not allow for drawing conclusions concerning the absolute levels of transcripts or proteins (POLR2A and H3 histone acetylated at Lys5), standardized methods and appropriate acquisition of fluorescent data let us to make comparative analyses between the two experimental series (control vs. HU). The obtained data indicate that under normal conditions, most of G1-phase cells display low fluorescence in the nucleoplasmic region. However, the number of cells with higher fluorescence increased in S- and G2 phase, and the intensity of fluorescence was enhanced 1.3-fold and 1.5-fold, respectively (Fig. 2). As compared with the control plants, HU-treated roots revealed the appearance of cells with significantly higher level of fluorescence intensity in every stage of interphase. Estimated values of fluorescence increased by average 1.8-fold in G1 phase, 1.9-fold in S phase, and 1.6-fold in G2 phase (Fig. 2).

**Fig. 3:**
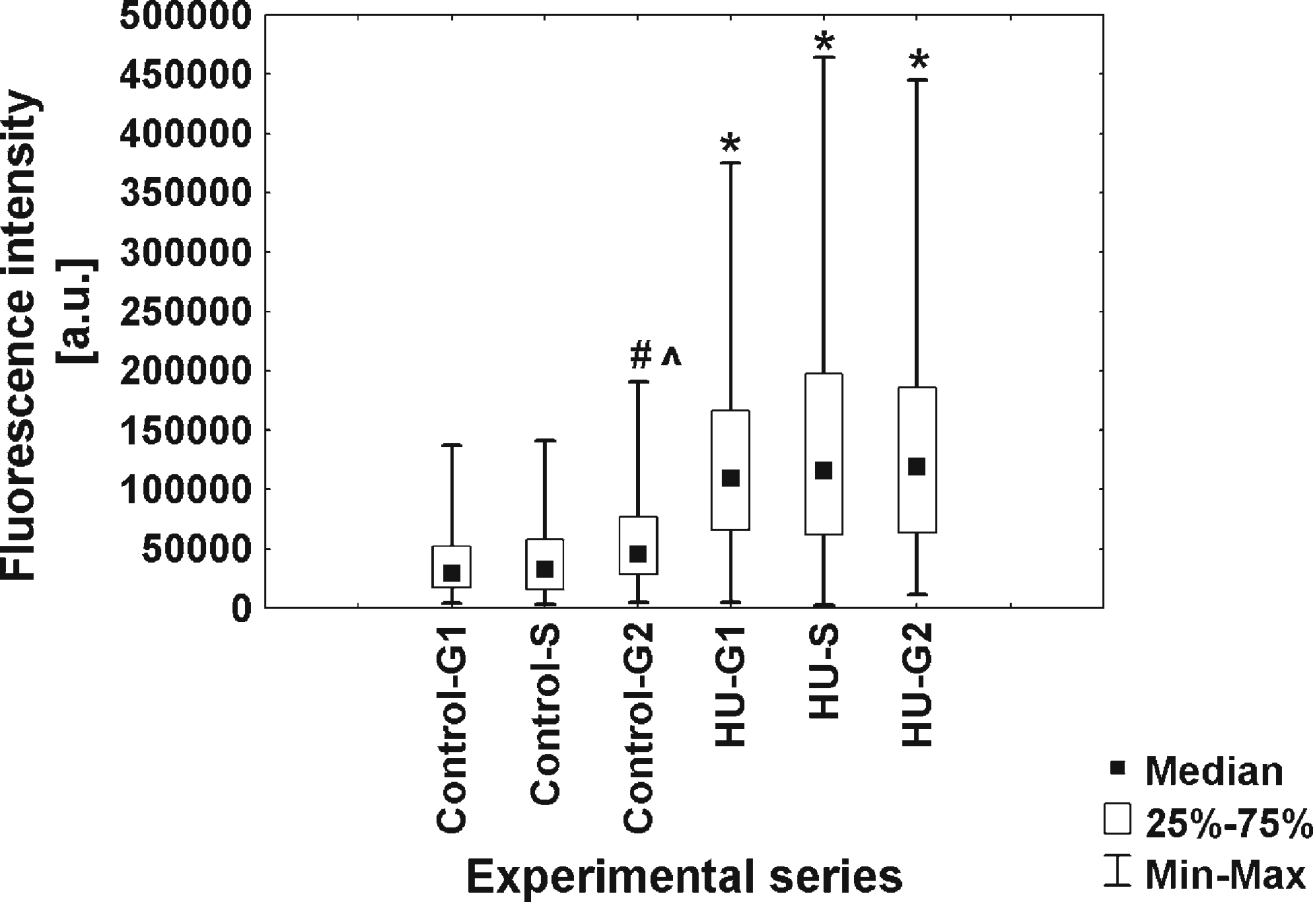
Median fluorescence intensity (*a.u.* arbitrary units) in the nucleoli evaluated following 5-EU incorporation into root tip cells from seedlings incubated in H_2_O and HU; successive phases of the cell cycle in the control plants denoted as *Control-G1*, *Control-S*, and *Control-G2* while in seedlings incubated in 2.5 mM HU (24 h) denoted as *HU-G1*, *HU-S*, and *HU-G2*. Statistical significance (Kruskal–Wallis test): *p < 0.001 control-G1/HU-G1, control-S/HU-S, control-G2/HU-G2; ^#^*p* < 0.001 control-G1/control-G2; ^^^*p* < 0.001 control-S/control-G2

**Fig. 4:**
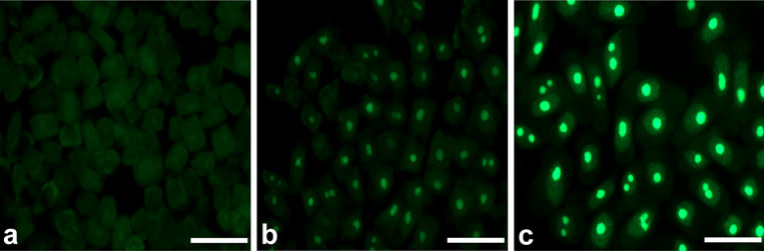
Cytochemical detection of transcription following 5-EU incorporation. **a** Negative control (without 5-EU), **b** incubation in H_2_O, **c** 24 h incubation with 2.5 mM HU. *Bar* 50 μm

**Fig. 5:**
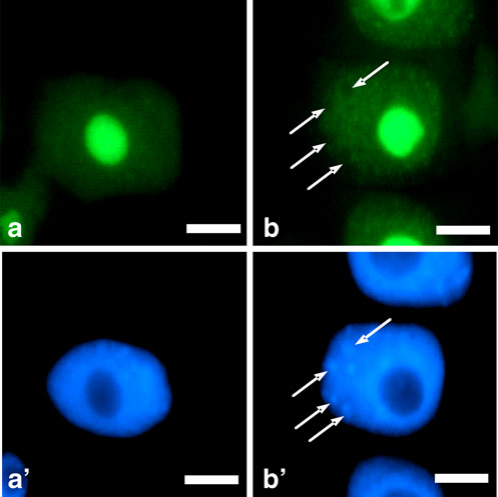
Selected cell nuclei showing intense 5-EU incorporation. **a** Incubation in H_2_O, **b** 24-h incubation with 2.5 mM HU; **a**’, **b**’ nuclei stained with DAPI. *Arrows* point to heterochromatic regions. *Bar* 10 μm

Under normal conditions, fluorescence in nucleoli remained constant throughout all stages of the cell cycle. However, in comparison with G1- and S phases, slight increase in the fluorescence intensity has appeared in the G2-phase cells (Fig. 3). In turn, the presence of HU enhanced the number of cells displaying higher fluorescence level. Median fluorescence intensity increased 3.7-fold in G1 phase, 3.5-fold in S phase, and 2.6-fold in G2 phase, in comparison with the control (Fig. 3). The observed changes in fluorescence clearly revealed an intensified transcription following HU treatment, both in the nucleoplasmic and nucleolar regions at every phase of the cell cycle (Figs. 4 and 5).

**Fig. 6:**
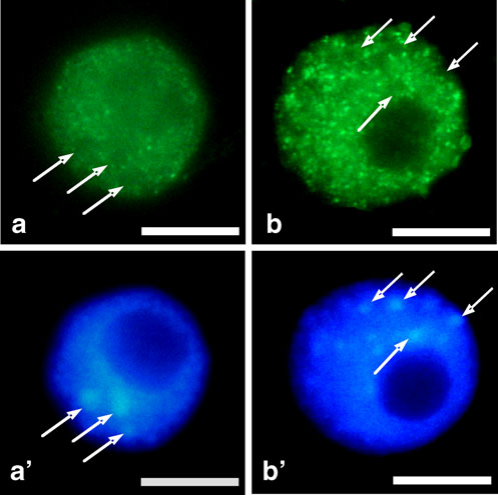
Immunocytochemical detection of polymerase RNA II large subunit (POLR2A). **a** incubation in H_2_0, **b** 24 h incubation with 2.5 mM HU; **a**’, **b**’ nuclei stained with DAPI. *Arrows* point to heterochromatic regions. *Bar* 10 μm

**Fig. 7:**
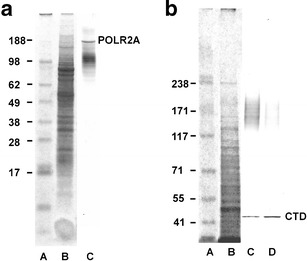
Immunoblotting analysis of POLR2A in whole-cell extracts (**a**) and nuclear protein lysates (**b**). Molecular weight marker (kilodalton; *lane A*), electrophoresis and Coomassie staining (*lane B*), extracts from control seedlings (*lane C*) and HU-treated seedlings (*lane D*) immunoblotted with anti-POLR2A (CTD) antibodies

Global character of transcription activation poses a question whether this process is accompanied by changes in RNA polymerase content or polymerase activity. High conservation within the C-terminal domains among human and plants enabled immunocytochemical identification of this protein by means of polyclonal antibodies against carboxyl-terminal domain (CTD) of the largest subunit of RNA polymerase II (POLR2A). Since immunofluorescence signal was restricted to euchromatin regions (heterochromatin and nucleoli were free of labeling), it can be assumed that the antibodies recognized the regions abundant in RNA polymerase II (Fig. 6). The specificity of antibodies has been tested using Western blot assay (Fig. 7). Following whole-cell protein extraction, a single band at 188 kDa was accompanied by a smear of lower molecular mass products (Fig. 7a). By using another procedure that allows for isolation of nuclear proteins, a single band has been found unexpectedly at a position corresponding to about 45 kDa (together with a fainter smear of higher mass products; Fig. 7b). Both Western blots suggest partial degradation of original POLR2A molecules and detected a 45-kDa peptide seems to be an equivalent of a CTD fragment derived from large subunit of RNA polymerase II (Kaneko and Manley 2005). Comparison between the lanes representing nuclear proteins extracted from the control and HU-treated roots indicates a slight increase in the 45-kDa peptide under stress conditions. Interestingly, previous papers reported POLR2A proteolysis during the procedure of protein isolation and consequently various electrophoretical mobility of peptides recognized by anti-POLR2A (CTD) antibodies (Guilfoyle et al. 1984; Armaleo and Gross 1985). Moreover, they point out an important problem concerning protein stability during preparation of extracts for Western blot analysis. Thus, improper conditions (e.g., pH of buffers) during isolation of some proteins may have an impact on the final interpretation of antibodies’ immunoreactivity.

**Fig. 8:**
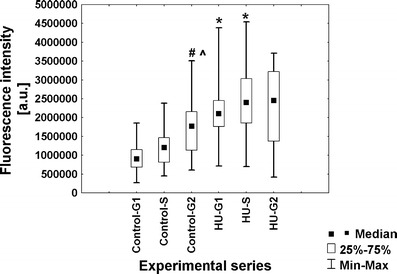
Median immunofluorescence intensity (*a.u.* arbitrary units) evaluated using anti-large subunit of RNA polymerase II (POLR2A) antibodies in seedlings incubated in H_2_O and HU. Successive phases of the cell cycle in the control plants denoted as *Control-G1*, *Control-S*, and *Control-G2*, while in seedlings incubated in 2.5 mM HU (24 h) denoted as *HU-G1*, *HU-S*, and *HU-G2*. Statistical significance (Kruskal–Wallis test): *p < 0.001 control-G1/HU-G1, *p < 0.001 control-S/HU-S; ^#^*p* < 0.001 control-G1/control-G2; ^^^*p* = 0.04 control-S/control-G2

The differences in immunoblotted protein level between the two experimental series have stimulated us to perform comparative immunofluorescence analyses at the cellular level. Quantitative analysis (Fig. 8) showed changes in POLR2A content during successive phases of the cell cycle under normal conditions (1.3-fold increase of fluorescence in S phase and 2.0-fold in G2 phase, in comparison with G1 phase). However, in comparison with the control, incubation of seedlings with HU triggered a 2.3-fold increase in median fluorescence intensity in G1 phase, 2.0-fold in S phase, and 1.4-fold in G2 phase. The obtained results clearly indicate that transcription activation following HU treatment is correlated with the transport of RNA polymerase II large subunit into cell nuclei.

Accumulation of POLR2A and enhancement of transcription in root meristems of *V. faba* seem to be consistent with some data indicating enhanced gene expression in response to HU treatment. Changes in transcription dynamics induced by HU did not concern stress-activated genes only (e.g., enhanced c-Fos expression; Yan and Hales 2005) but also comprise: (a) an increase in human γ-globin mRNA level (Watanapokasin et al. 2006), (b) expression of adult β-globin gene in HeLA cells (Zhang et al. 2001), (c) enhanced *SMN2* (survival motor neuron) gene expression in lymphoblastoid cell lines (Grzeschik et al. 2005), and (d) accumulation of cyclin B-like proteins in *A. cepa* (Żabka et al. 2011). Moreover, other genotoxic stresses (such as ionizing radiation) triggered multiple gene activation in *Caenorhabditis elegans* (Greiss et al. 2008). However, since the majority of genes are not related to DNA damage checkpoints and DNA repair, the above authors suggest that most of them might be related to general stress responses. The obtained results are in contrary to those presented by Cui et al. (2010), who revealed transcription suppression in response to HU treatment of mouse embryonic stem cells. Taking into account the global character of transcription activation comprising both nucleoli and nucleoplasmic regions in root meristem cells of *V. faba*, as well as other data, it seems reasonable to conclude that not only stress response-specific genes might be activated in *V. faba* following HU treatment. However, this hypothesis still needs to be proved.

### Hydroxyurea triggers changes in histone H4 acetylation

**Fig. 9:**
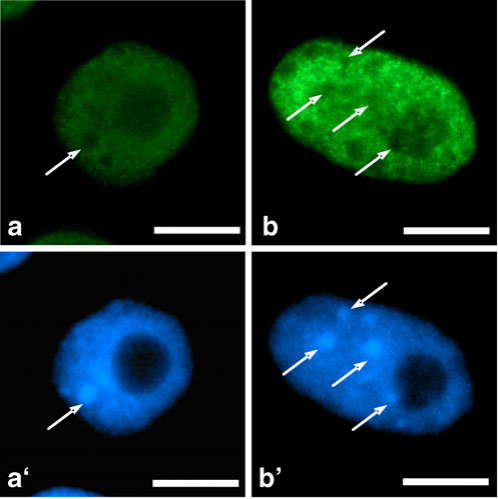
Immunocytochemical detection of histone H4 Lys5 acetylation. **a** Incubation in H_2_0, **b** 24-h incubation with 2.5 mM HU; **a**’, **b**’ nuclei stained with DAPI. *Arrows* point to heterochromatic regions. *Bar* 10 μm

**Fig. 10:**
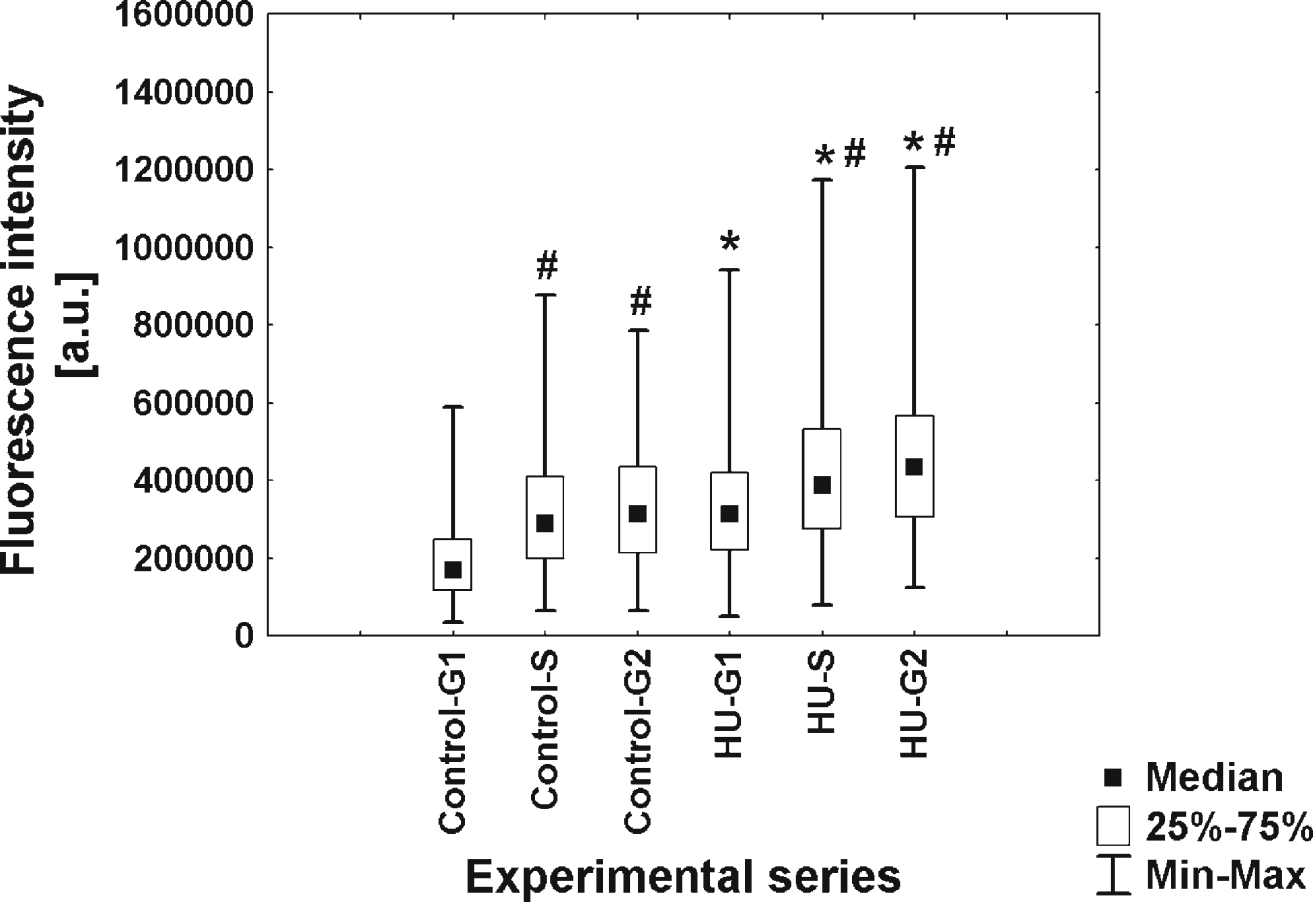
Median immunofluorescence intensity (*a.u.* arbitrary units) evaluated following detection of histone H4 acetylated Lys5 in seedlings incubated in H_2_O and HU. Successive phases of the cell cycle in the control plants denoted as *Control-G1*, *Control-S*, and *Control-G2*, while in seedlings incubated in 2.5 mM HU (24 h) denoted as *HU-G1*, *HU-S*, and *HU-G2*. Statistical significance (Kruskal–Wallis test): *p < 0.001 control-G1/HU-G1, control-S/HU-S, control-G2/HU-G2; ^#^*p* < 0.001 control-G1/control-S, control-G1/control-G2, HU-G1/HU-S, HU-G1/HU-G2

Since HU treatment led to transcription activation and to an increase in POLR2A content in cell nuclei of *V. faba*, one could ask whether this response is correlated with posttranslational modifications of histones that play a crucial role in opening up chromatin. To resolve this problem, we have concentrated on immunodetection of H4 histone acetylated at Lys5 (Fig. 9), which in *V. faba* was previously performed by Belyaev et al. (1997) and Jasencakova et al. (2000). Under normal conditions, the number of cells displaying intense fluorescence rose during cell cycle progression (in comparison with G1 phase, level of immunofluorescence increased 1.7- and 1.9-fold in S- and G2 phase, respectively; Fig. 10). These data are consistent with earlier reports indicating histone H4 Lys5 acetylation in TALO8 minichromosome system throughout S and G2/M phases (Unnikrishnan et al. 2010) and prior deposition of histone H4 onto DNA during replication (Sobel et al. 1995; Chang et al. 1997; Benson et al. 2006). Furthermore, HU treatment of primary roots resulted in the appearance of cells with higher fluorescence in every phase of cell cycle. In comparison with the control plants, median fluorescence intensity increased 1.9-fold in G1 phase, 1.4-fold in S phase, and 1.4-fold in G2 phase (Fig. 10).

A high level of H4 Lys5 acetylation in HU-treated seedlings indicates that global transcription activation depends on histone modifications. This result seems to be consistent with that presented by Sharma et al. (2007), who showed an increase in H4 acetylation and recruitment of RNA polymerase II to *RNR3* and *HUG1* promoters in response to DNA-damaging agent, methyl methanesulfonate. Moreover, in *Saccharomyces cerevisiae*, enhanced Lys5 acetylation can be found in soluble fraction of histone H4 following HU treatment (Poveda and Sendra 2008) and at sites of DSB (Tamburini and Tyler 2005). However, McCaffrey et al. (1997) showed that HU does not affect histone acetylation in human K562 cells. In turn, Jasencakova et al. (2000) link H4 Lys5 acetylation in *V. faba* with replication rather than transcription. Although these data seem to be in contrast to the results presented in our paper, Belyaev et al. (1997) clearly show that unlike euchromatin, late-replicating heterochromatic regions of *V. faba* metaphase chromosomes are hypoacetylated, and only roots treated with trichostatin A (deacetylase inhibitor) display acetylation of heterochromatic regions in metaphase chromosomes. Therefore, obtained data still let us to assume that observed acetylation is associated with transcription, at least in the G1- and G2 phases. Furthermore, taking into account previous results of Sobel et al. (1995) and Benson et al. (2006) and those presented in our paper, it cannot be ruled out that in plants H4 Lys5 acetylation plays a role both in transcription and replication. Thus, it is reasonable to assume that acetylation observed in euchromatin during S phase may determine regions competent both for gene expression and ongoing nucleosome assembly throughout DNA synthesis.

## Conclusions

Taking into account the presented results, one may ask which cellular pathways modulate transcription activation in the cells exposed to HU. Some hypotheses relevant to this problem have appeared after analyses performed using animal models. HU generates reactive oxygen species (ROS), e.g., hydrogen peroxide and nitric oxide, which induce activation of diverse genes (Sakano et al. 2001). Enhanced transcription might be triggered by mitogen-activated protein (MAP) kinase signaling pathway since ROS were found capable to activate p38 kinase in animals (Huwiler and Pfeilschifter 1999; Jia et al. 2007). However, recent analyses have shown that an increase in H4K5 acetylation following HU treatment of *V. faba* was reduced by applying caffeine, an inhibitor of ATM/ATR kinases (Krajewski and Maszewski, in preparation). This may indicate that both ROS and the presence of DNA lesions could directly trigger changes in transcription dynamics. In plants, this kind of ATM/ATR-mediated transcription activation in response to DSBs might also involve MAP kinase pathway since in animals following cisplatin-, doxorubicin-, or camptothecin-induced DNA damages, ATM/ATR activate p38MAPK/MK2 (Reinhardt et al. 2007). Moreover, p38 kinase was reported to be activated following HU treatment and, together with Chk1, responsible for the block of mitotic entry (Rodríguez-Bravo et al. 2007). It seems thus that a more detailed examination of the activated genes and the potential mechanisms underlying response to HU still needs to be done, especially when considering HU as a therapeutic agent.
